# American Medical Association

**Published:** 1874-07-01

**Authors:** N. S. Davis

**Affiliations:** Chairman of Committee; Detroit


### AMERICAN MEDICAL ASSOCIATION.

### Report of Judicial Council.

DR. N. S. DAVIS, of Illinois, Chairman of the Judicial Council, read the following report:

The undersigned Committee, to whom was referred the question of a general revision of the Code of Ethics of the American Medical Association, respectfully report as follows: Soon after the adjournment of the annual meeting in May, 1873, the Chairman of your Committee, being desirous of ascertaining how far there existed a desire in the minds of the profession to have the Code changed, addressed letters of inquiry to thirty or forty men in different parts of the country, who might be supposed to represent the general sentiment of the profession in their respective districts, from twenty-five of whom answers were received. Fourteen expressed an entire approval of the Code as it is, and thought it better to attempt no changes. Six were opposed to any general revision, but supported slight changes in some sections, while only five expressed such dissatisfaction as to indicate a desire for thorough revision.

If these correspondents might be regarded as fairly representing the profession, any important changes in the Code would be in direct opposition to the wishes of a large majority of those for whose guidance it was framed. Those who have expressed a desire for changes in the present Code are readily divided into two classes. The first class appear to look upon a Code of Ethics in the same light as ordinary By-Laws, and consequently regard all in the present Code, relating to the duties of patients to the physician, of the public to the profession, and of the profession to the public, as superfluous and useless. They would retain nothing except the rules governing the intercourse of physicians with each other, and a very few of this class object to any *written* rules, claiming that the unwritten sense of *honor* belonging to members of an educated profession is sufficient to afford all needed guidance and control. It seems to us that the objections of this whole class are founded on a narrow and imperfect conception of the real nature and objects of an Ethical Code. The latter, instead of consisting of a set of rules or By-Laws, simply defining the duties and privileges of members of some organized Society, should be a concise and full exposition of the relations sustained by a profession to the rest of the community, the mutual obligations imposed by such relations, and the rules governing members in their intercourse with each other. Hence, a Code of Ethics for our profession must partake more or less of the nature of a moral essay, developing principles for guidance equally applicable to all places and times, instead of a few simple rules applicable to the members of some particular Society. It was with this view that Dr. Percival wrote his celebrated essay in the latter part of the last century, and which has been regarded as a standard authority in Europe from that time to the present. The same idea evidently controlled the very able Committee appointed by the preliminary convention in 1846 to report a Code of Ethics for the profession of this country, and who gave us the admirably concise and well arranged summary of the principles evolved by Percival, which constitutes our present Code.

To strike out, as some have proposed to do, all relating to the duties of patients, the community, and the public toward the profession, would be to destroy the completeness of the work, and obscure the meaning of what was retained. For the members of any given profession cannot rightly appreciate the relations they sustain to each other, without considering at the same time their mutual relations and duties to the community in which they live. After a very careful examination, we are satisfied that the present Code of Ethics presents the true ethical relations and duties of the profession, in a form as concise, as well arranged, and as complete as it is possible to express them. The principles and rules of conduct enumerated are clearly stated, and are equally applicable for the guidance of all who attempt to practice the healing art, whether they are members of any medical organization or not.

The second class, whose members desire alterations in the present Code of Ethics, do not object to its general scope, but ask for amendments or additions to particular sections only. With only a few and unimportant exceptions, these propositions all relate to two subjects, namely, *specialties* and bestowing professional services by *contract*. Concerning the first of these, there seems to be much false reasoning and needless irritation. If *specialists*, or those who limit their practice to some one class of diseases or accidents, are members of the profession, it follows, logically, that they must be governed in all respects by the same ethical principles as the general practitioners; for no enlightened body of men can consistently have one Code of morals for one part of its members and another Code for the rest. Whatever may be regarded as derogatory to the dignity and welfare of the general practitioner, must be equally so to the specialist so long as he is recognized as a member of the profession. If the one may not issue cards, hand-bills, etc., calling the attention of those laboring under particular diseases to themselves, neither can the other, without violating the principles of both justice and equality.

The Code of Ethics very properly makes no mention of specialties or specialists, but presents plainly the rules necessary for the maintenance of professional character as applicable to all. But we are asked, how, then, can those who wish to pursue a special practice make known their position to their brethren and the public? We answer, that the title of Doctor of Medicine covers the whole field of practice, and whoever is entitled to the appellation has the right to occupy the *whole* or any part of the field, as he pleases. The acceptance of this honorable title is presumptive evidence to the community, that the man accepting it is ready to attend practically to any and all duties which it implies. As all special practice is simply a self-imposed limitation of the duties implied in the general title of doctor, it should be indicated, not by special or qualifying titles, such as *occulist*, *gynecologist*, etc., nor by any positive setting forth of special qualifications, but by a simple, honest notice appended to the ordinary card of the general practitioner, saying, "Practice limited to diseases of the eye and ear," or "to diseases peculiar to women," or "to midwifery exclusively," as the case may be. Such a simple notice of limitation, if truthfully made, would involve no other principle than the notice of the general practitioner that he limits his attention to professional business within certain hours of the day. Neither could it be regarded as a claim to special or superior qualifications. To give the specialist any privilege beyond this, would be to invest him with a special privilege inconsistent with the equality of rights and duties pertaining to the whole profession. We see no reason, therefore, for recommending any change in the present Code of Ethics in reference to this subject.

The remaining topic, concerning the bestowal of professional services under specific *contracts* specifying the amount of pecuniary compensation, is of sufficient importance to require careful attention.

The present Code of Ethics, while sanctioning a most liberal bestowal of gratuitous professional service to the poor, whether as individuals or in public charitable institutions, and in aid of the sanitary interests of communities, yet expressly prohibits the bestowal of such services on well-to-do individuals, endowed, mutual benefit, or any kind of money-making institutions, societies or corporations. It also expressly prohibits all attempts to attract attention or make merchandise of charity by ostentatiously parading before the public notices proffering services and medicine to the "poor gratis." We see no reason why this is not sufficient so far as relates to the regulation of gratuitous services. To govern the matter of compensation, the Code simply gives us the following general declaration: "Some general rules should be adopted by the *faculty*, in every town or district, relative to *pecuniary acknowledgments* from their patients; and it should be deemed a point of honor to adhere to these rules with as much uniformity as varying circumstances will admit." The aim appears to have been, to allow sufficient variations in the rate of compensation to accommodate the varying habits and circumstances of different communities, and yet to bind each individual to an honorable compliance with the general rules established by his professional brethren. Such being the correct ethical principle, the difficulty consists in tracing and maintaining clearly its practical application. That the principle laid down in the paragraph just quoted is inconsistent with all contracts or agreements to attend individuals, families, companies, corporations, or any associations or institutions other than those of a strictly charitable character, for a specified sum per month or year, without regard to the amount of medical services that might be required in the time specified, no one can reasonably doubt. It seems to us equally inconsistent with the ethical rule to enter into a contract with a manufacturing company to attend their employes, or with a school to attend its patrons or scholars, for a fixed sum per annum, to be derived from the levy of a certain per centage on the wages of the employes or on the tuition fees of the students; for however plausible may be the humanitarian idea of securing for the employe and student adequate medical attendance when sick at the smallest average cost, the practical working of the system violates both the rule that compensation for medical services should be in accordance with the kind and amount of services rendered, and that every individual and family should be free to choose their own medical attendant without dictation or indirect restraint.

These observations do not apply to a certain kind of contract service, sometimes required in connection with the medical staffs of the army and navy, nor to the hospital tax on sailors in the marine hospital system, for reasons too obvious to require mention. One other subject requires a few moments attention. There is a class of public charitable institutions, such as county almshouses, orphan asylums, etc., supported by public taxation. In many of the States the public authorities having control of such institutions, have annually asked for bids from the profession, offering to award the contract for professional services to the one who should bid for the lowest pecuniary consideration. While as charitable institutions any member of the profession might offer his services to such of the poor inmates as might ask for them, gratuitously; yet the idea of asking members of the profession to bid against each other, for the pay for public professional services, is repugnant to every feeling of professional honor, and often productive of great injustice to the sick poor.

The public authorities in all such cases should fix just rate of compensation for the necessary medical services as they may deem best, and then appoint the best medical man who is willing to accept the compensation proposed. And, we have no doubt, but that a proper attention to this subject on the part of the profession would secure the necessary change.

It is, however, very desirable to so manage all our pecuniary relations with the public, and especially with municipal and legislative authorities, that we avoid creating the impressions on the public mind that the profession and its social organizations are little better than mere trades-unions, having for their chief object mutual pecuniary protection. After carefully reviewing the whole subject, your Committee do not recommend any alteration in the present Code of Ethics. On the contrary, we desire to express the opinion that if every medical school and society would supply each graduate as he left the school, and each member initiated into the society, with a printed copy of the Code, accompanied with the injunction that it be carefully studied, it would be productive of much good, directly to the profession and indirectly to the community.

N. S. DAVIS,
*Chairman of Committee.*

Detroit, June 3, 1874.

